# A new contender enters the race for cystic fibrosis gene therapy

**DOI:** 10.1016/j.omtn.2026.103146

**Published:** 2026-11-04

**Authors:** Laudonia Lidia Dipalo, Kasper Gryspeert, Marianne S. Carlon

**Affiliations:** 1 Department of Chronic Diseases and Metabolism, Laboratory of Respiratory Diseases and Thoracic Surgery (BREATHE), KU Leuven, Leuven, Belgium

## Main text

As a monogenic disorder, cystic fibrosis (CF) has long been viewed as an attractive candidate for a one-size-fits-all gene therapy approach. Combined with the fact that the most clinically relevant target cells are located within the human airway epithelium (HAE), which was once thought to be readily accessible through aerosolized vector delivery, CF appeared to be the ideal disease for targeted gene therapy development. However, except for two currently ongoing studies, all clinical trials for viral vector-mediated, lung-targeted *CFTR* gene therapy have been stopped in their tracks before phase 3. The multifaceted underlying reasons include the difficulty to transduce mature human airway epithelial cells (HAECs), which have been honed by evolution to act as barriers against invading pathogens, and the inability to reach sufficient levels of CFTR expression throughout the HAE.^1^ In the previous issue of *Molecular Therapy Nucleic Acids*, Phillips, Lei et al. recently proposed a novel gene delivery platform that may circumvent both issues. Their vector achieved efficient transduction of mature HAECs in a clinically relevant CF airway model, remarkably leading to full correction of CFTR ion channel function.^2^ Nevertheless, further studies may be needed to assess the durability of CFTR expression and to identify the cell types responsible for the functional rescue.

Most of the viral vectors commonly used in gene therapy have tried tackling CF lung disease, but none have been successful to this day. Nine adenoviral vector-based clinical trials for CF gene therapy were initiated between 1993 and 2001, whereas recombinant adeno-associated viral vectors (rAAVs) have dominated the field since 2002, with a rAAV2-*CFTR* vector marking the first ever rAAV-based human clinical trial. Disappointingly, all trials eventually came to a halt due to high immunogenicity and/or insufficient transgene expression, except for two rAAVs currently under clinical investigation. The first-in-man lentiviral vector (LV) clinical trial for CF, leveraging the Sendai-H/FN envelope proteins for lung targeting, was launched in late 2024 and terminated in early 2026 prior to concluding the trial, hence no results have been communicated on primary endpoint outcomes (Table 1).^1^^,^^3^

**Table 1:** Landscape of clinical trials for lung-targeted gene therapy for cystic fibrosis

*CFTR* gene therapy product	Delivery mode	Outcome of the study
**Non-viral delivery**		

Cationic liposome composed of lipids DC-Chol and DOPE (3:2 molar ratio) complexed with pDNA containing the CMV promoter sequence alone (in the tolerability study) or driving the expression of WT *hCFTR* cDNA (in the phase 1/2 trial)	Nasal nebulization of 10 equal doses administered every 15 min for a total of 135 min	**Tolerability study (results released in 1994/07)** no SAEs nor airway physiology modifications recorded
		**Phase 1/2 trial (results released in 1995/01)** no SAEs recorded; nasal PD restored up to 20% of physiological values; correction peaked at day 3 and was fully reverted by day 7 post-treatment
Cationic liposome composed of lipids DC-Chol and DOPE (3:2 molar ratio) complexed with the pTRIAL10CFTR2 plasmid optimized for WT *hCFTR* cDNA expression	Nasal instillation of a single dose spread in 4 sessions over the course of 2 days	**Phase 1 trial (results released in 1997/03)** no SAEs recorded; nasal PD restored within physiological range for up to 15 days post treatment
	Nasal instillation of three doses with 4-week intervals	**Repeated dosing study (results released in 2000/07)** no SAEs recorded; no statistically significant difference in nasal PD pre- and post- treatment
*pCMV-CFTR-DOTAP*: cationic liposome composed of the lipid DOTAP complexed with the pCMV-*CFTR* plasmid (1:6 weight ratio)	Intermittent nasal nebulization over a 2 h period	**Phase 1 trial (results released in 1997/03)** no SAEs recorded; no statistically significant difference in nasal PD pre- and post- treatment
Cationic liposome composed of lipids GL-67 and DOPE (1:2 molar ratio) complexed with the plasmid pCF1-*CFTR* encoding WT *hCFTR* cDNA under the regulation of the CMV promoter/enhancer and the Ad2 tripartite leader (1.6:1 lipid:DNA weight ratio)	Endoscopically guided nasal instillation over an 80 min period	**Phase 1 trial (results released in 1997/09)** no SAEs recorded; both DNA alone and lipid-DNA complex partially restored normal Cl^−^ secretion in the nasal epithelium for ≥4 days post-treatment
pCF1-*CFTR* plasmid alone		
Cationic lipid formulation GL-67 A (lipids GL-67, DOPE, and DMPE-PEG_5000_ complexed at molar ratio 1:2:0.05) complexed with the plasmid pGM169 encoding WT *hCFTR* cDNA under the regulation of the CMV promoter (5:1 lipid:DNA weight ratio)	Lung-directed (1^st^ dose) and nasal (2^nd^ dose, 7 days later) nebulization	**Phase 1 trial (results released in 1999/03)** no SAEs but mild flu-like symptoms recorded; CFTR secretory function restored to ∼20% and ∼25% of physiologic values in nasal and bronchial epithelium, respectively
	Lung-directed nebulization	**Phase 1/2a repeated dosing trial (n°**NCT00789867**, 2009/08–2009/08,** **results released in 2015/12)** no SAEs recorded, but flu-like symptoms experienced by ∼ all patients; no improvement of nasal PD, but increased Cl^−^ secretion up to 14 days post-treatment
	Lung-directed nebulization of 12 doses administered at intervals of 28 ± 5 days	**Phase 2b repeated dosing trial (n°**NCT01621867**, 2012/05–2014/05,** **results released in 2016/07)** no SAEs recorded; modest but statistically significant improvement in FEV_1_ and Cl^−^ secretion, reported to not improve the patients’ quality of life
Cationic liposome composed of lipids EDMPC and cholesterol, and the plasmid MB113, encoding a CMV promoter driving WT *hCFTR* cDNA expression (1:2:8 molar ratio)	Intermittent nasal nebulization over a 7 h period	**Phase 1 trial (results released in 2000/01)** no SAEs recorded; no statistically significant difference in nasal PD pre- and post- treatment
Cationic liposome composed of a plasmid encoding WT *hCFTR* cDNA and the lipid GL-67	Intranasal instillation	**Dose escalation study (n°**NCT00004806**, results released in 2000/01)** SAEs recorded in 50% of the patients
Compacted DNA nanoparticles composed of the plasmid pKCPIRCFTRBGH, encoding the WT *hCFTR* cDNA under the regulation of the CMV promoter/enhancer, and polyethylene glycol-substituted lysine 30-mer peptides	Instillation in the nasal cavity	**Phase 1 dose escalation trial (results released in 2004/12)** no SAEs recorded; partial correction of Cl^−^ transport was detected, peaking between 1 and 7 days post-treatment
*ARCT-032:* WT *hCFTR* mRNA packaged in a proprietary LUNAR lipid nanoparticle formulation	Lung-directed nebulization	**Phase 1/2 trial (n°**NCT06747858**“Lunair-CF,” 2022/12 - ongoing)** • **phase** **1** **interim results released in 2024/10** no SAEs recorded; trends toward mucus plug reduction were observed; phase 2 currently ongoing
*VX-522:* WT *hCFTR* mRNA packaged in a proprietary lipid nanoparticle formulation	Lung-directed nebulization	**Phase 1/2 trial (n°**NCT05668741**, 2023/02–2026/05)** terminated before completion due to tolerability issues
*RTC2100*: WT *hCFTR* mRNA packaged in a proprietary SORT lipid nanoparticle formulation	Lung-directed nebulization	**Phase 1/2 multicentre trial (n°**NCT06237335**, 2024/02 – ongoing)** phase 1 concluded with no SAEs recorded; phase 2 ongoing, no data available yet
*BMD003*: non-lipid nanoparticle formulation composed of freeze-dried WT *hCFTR* mRNA molecules	Lung-directed nebulization	**Phase 1 trial (n°**NCT06963008**for adult patients, 2025/26 – ongoing, and n°**NCT07485543**for patients ≥ 12 yo, 2026/04 - ongoing)** no data available yet

**Viral-based delivery: recombinant Adenoviral vectors (AdVs)**		

*Ad2/CFTR-1:* replication-deficient, E1-deleted rAdV2 harboring the WT *hCFTR* cDNA sequence	Nasal nebulization	**Phase 1 trial (results released in 1993/10)** no SAEs, but mild inflammatory responses recorded; nasal PD restored to physiological range for up to 3 weeks post-treatment
*AdCFTR:* replication-deficient, E1a-, partial E1b-, and partial E3- deleted rAdV5 harboring the WT *hCFTR* cDNA sequence under the regulation of the AdV2 major late promoter and tripartite leader	Nasal (1^st^ dose) and bronchoscopic (2^nd^ dose, 1 day later) instillation	**Phase 1a trial (results released in 1994/09)** no SAEs, but mild inflammatory responses recorded
		**Phase 1b trial (results released in 1995/11)** no SAEs, but mild inflammatory responses recorded; nasal PD restored to ∼33% of physiological levels at 14 days post-treatment
*Ad5-CB-CFTR or H5.020CBCFTR*: replication-deficient, E1- and partial E3- deleted rAdV5 harboring the WT *hCFTR* cDNA sequence under the regulation of a CMV enhancer and chicken beta-actin promoter	Bronchoscopic instillation	**Tolerability study (results released in 1995/09)** absence of toxicity and detectability of vector DNA up to 8 days post-treatment confirmed
	Nasal instillation	**Phase 1 pilot trial (n°**NCT00004779**, results released in 1995/09)** no SAEs recorded; transduction efficiency of the nasal epithelium ≤1%, with no significant change in nasal PD pre- and post- treatment
*Ad2/CFTR-2:* replication-deficient, E1- and partial E4- deleted rAdV2 harboring the WT *hCFTR* cDNA sequence under the regulation of a PGK promoter	Nasal instillation of 5 escalating doses	**Repeated dosing study (results released in 1996/01)** no SAEs, but mild inflammatory responses recorded; partial correction of nasal PD, with high inter-patient variability
*AdCFTR*: replication-deficient, E1a-, partial E1b-, partial E3- deleted rAdV5 harboring the WT *hCFTR* cDNA sequence under the regulation of an AdV2 major late promoter and tripartite leader	Nasal instillation and aerosolization to the lungs	**Phase 1 trial (results released in 1997/01)** no SAEs recorded; low levels of WT *hCFTR* mRNA detectable in patient-derived samples for up to 15 days post-treatment
*Ad*_*GV*_*CFTR.10:* replication-deficient, E1a-, partial E1b, and partial E3- deleted rAdV5 subgroup C harboring the WT *hCFTR* cDNA sequence under the regulation of a CMV promoter and enhancer sequence	Endobronchial aerosolization of 3 doses over the course of 9 months via bespoke spray delivery system	**Phase 1 trial (results released in 1999/11)** no SAEs recorded; low levels (∼5% of physiological) of WT *hCFTR* mRNA detected in patient-derived samples at 3 days post-treatment, but no longer detectable at 30 days; 2^nd^ dose yielded modest and variable increase in efficiency; 3^rd^ dose yielded no additional benefit
*H5.001CBCFTR*: replication-deficient, E1- and E4- deleted rAdV5 harboring the WT *hCFTR* cDNA sequence under the regulation of a CMV enhancer and chicken beta-actin promoter sequence	Bronchoscopic instillation	**Phase 1 trial (n°**NCT00004287**, results released in 1999/12)** no SAEs but mild inflammatory responses recorded; transduction efficiency in bronchial epithelium ≤1%; no correction of Cl^−^ transport
*Ad2/CFTR8:* replication-deficient, E1- and E4- deleted rAdV2 harboring the WT *hCFTR* cDNA sequence under the regulation of the AdV2 E1a promoter	Nasal nebulization or bronchoscopic instillation	**Phase 1 trial (clinical protocol CF94-0401, results released in 2001/06)** no SAEs recorded; transduction efficiency of ∼2.4% and ≤1% in nasal and bronchial epithelial cells, respectively; WT *hCFTR* mRNA detected in patient-derived samples up to 7 days post-treatment

**Viral-based delivery: recombinant adeno-associated viral vectors (rAAVs)**		

*tgAAV-CFTR:* rAAV2 harboring the WT *hCFTR* cDNA sequence under the regulation of the native adeno-associated viral promoter sequence	Nasal instillation	**Phase 1 trial (results released in 1999/02)** no SAEs recorded; partial correction of nasal PD
		**Phase 1 trial (results released in 2002/07)** no SAEs recorded; no statistically significant nasal PD correction
	Nasal nebulization	**Phase 2a trial (results released in 2002/10)** 3 SAEs recorded in a 12-patient cohort; WT *hCFTR* mRNA expression was not detected
	Nasal nebulization of 3 doses delivered at 30-day intervals	**Phase 2a trial (results released in 2004/02)** no SAEs recorded; statistically significant improvement in FEV_1_ detected at day 30, but not day 60 post-treatment
	Nasal nebulization of 2 doses delivered at 30-day intervals	**Phase 2b multicenter trial (n°**NCT00073463**2003/06–2005/10,** **results released in 2007/08)** no SAEs recorded; primary efficacy endpoint on FEV_1_ improvement was not met
	Nasal (1^st^ dose) and bronchoscopic (2^nd^ dose, 1 day later) instillation	**Phase 2 trial (n°**NCT00004533**, results released in 2003/06)** no SAEs but mild inflammatory responses recorded
*4D-710*: proprietary mucus-penetrating, rAAV2-derived A101 capsid harboring the WT *hCFTRΔR* minigene under the regulation the CMV173 promoter sequence	Lung-directed nebulization	**Phase 1/2 multicenter trial (n°**NCT05248230**, 2022/03 – ongoing)** • **phase 1 interim results released in 2022/10** no SAEs recorded; *hCFTRΔR* mRNA detected in ≥40% bronchial epithelial cells within patient-derived samples
*SP-101*: proprietary airway-tropic AAV2.5T capsid (AAV2/AAV5 hybrid) harboring the WT *hCFTRΔR* minigene under the regulation of the SP183 synthetic promoter/enhancer sequence	Lung-directed co-nebulization with transduction-augmenter doxorubicin	**Phase 1/2 multicenter trial (n°**NCT06526923**, 2024/09 – ongoing)** no results released yet

**Viral-based delivery: others**		

*KB407:* replication-defective, non-integrating herpes simplex virus type 1 (HSV-1)-derived vector harboring WT *hCFTR* cDNA	Lung-directed nebulization of ascending and/or repeated doses	**Phase 1 trial (n°**NCT0550483**, 2023/06 – ongoing)** • **tolerability interim results released in 2024/12** no SAEs recorded • **efficacy interim results released in 2026/09** transduction efficiency ∼36% in bronchial epithelial cells within patient-derived samples
*BI 3720931*: 3^rd^ generation lentiviral vector pseudotyped with Sendai virus F and HN envelope proteins (rSIV.F/HN) and harboring WT *hCFTR* cDNA under the regulation of a hybrid, CpG-free promoter sequence consisting of the EF1a promoter and the CMV enhancer	Lung-directed nebulization via bespoke inhaler	**Phase 1/2 trial (n°**NCT06515002**, 2024/11–2026/02)** terminated before completion due to sponsor decision; no data on safety and efficacy were released

An extensive list of clinical trials for lung-targeted gene therapy for cystic fibrosis is provided, subdivided by delivery vector type and chronologically ordered. Trials that have been concluded or terminated for over 3 years with no results released on safety or efficacy have been excluded. The data was compiled on the basis of the references listed in Plasschaert et al.^1^ as well as by searching the ClinicalTrials.gov NIH database.^3^

WT *hCFTR*, wild-type human cystic fibrosis transmembrane conductance regulator; rAdV, recombinant adenoviral vector; rAAV, recombinant adeno-associated viral vector; SAEs, severe adverse events; nasal PD, nasal potential difference; FEV_1_, forced exhalation volume in 1 s; Cl^−^, chloride anion.

Preclinically, other viral vector candidates have been explored. A notable example is Zhang et al.’s replication-competent, human parainfluenza virus 3 (PIV3)-based vector, which achieved ∼25% transduction efficiency and full correction of CFTR ion channel function *in vitro*, but caused significant cytotoxicity due to its pathogenic nature in humans.^4^ Phillips, Lei et al. move the focus toward PIV5—a close, non-pathogenic relative of PIV3. In 2017, Wei, Chen, and colleagues developed PIV5-like vectors which, while incapable of viral replication, retained the machinery responsible for intracellular amplification of the episomal viral genome and embedded transgenes—hence termed self-amplifying virus-like particles (AVLPs).^5^

Building further on this, McCray’s team elegantly translated PIV5 AVLPs into a lung-tropic *CFTR* cDNA delivery vehicle. Owing to their single-stranded RNA genome, PIV5 AVLPs drive transient transgene expression with virtually no risk for genomic integration, while the self-amplification machinery may partially compensate the progressive dilution of non-integrating viral genomes in replicating cells—an interesting compromise between the perks of LVs and rAAVs. Further, their large genome—∼15 kb, minus the sequences encoding the self-amplification machinery—enable PIV5 AVLPs to shuttle genetic payloads ∼15%–20% larger than those carried by rAAVs.^2^ Only apparently minute, this difference unlocks the possibility of packaging a full-length, codon-optimized *CFTR* cDNA rather than the truncated *CFTR* minigene optimized to fit within the restrictive cargo capacity of traditional rAAVs.^1^ Impressively, PIV5 AVLPs transduced ∼15% HAECs *in vitro* without co-administration of any pre-treatment to disrupt the epithelial tight junctions—a common practice which, while proven both reversible and effective in animal models, may allow the pathogens responsible for chronic airway infections in CF to penetrate the epithelial barrier. Following transduction, CFTR-dependent short-circuit currents were restored to wild type (WT)-like levels measured in non-CF HAEs, in line with previous reports suggesting that 5%–10% and 10%–25% of CFTR-expressing cells are sufficient to ameliorate and abolish, respectively, CFTR ion channel dysfunction.^1^^,^^2^

The majority of transduced cells were ciliated (∼70%), followed by secretory (∼23%) and basal (∼10%) cells.^2^ The transduction pattern roughly mirrors the relative abundance of each cell type in native airway epithelia,^6^ suggesting that transduction occurred in a non-cell-type-specific manner. An unanswered question to date is whether ciliated cells, physiologically hardly expressing any CFTR, are able to support CFTR ion channel activity from ectopic *CFTR* gene expression. In 2007, Ostrowski et al. showed that ciliated cell-specific expression of human CFTR restored cAMP-stimulated chloride secretion in neonatal CF mouse tracheas but failed to normalize Cl-driven nasal potential differences in adult mice despite robust apical CFTR expression in ciliated cells. They interpreted that additional epithelial cell types contribute substantially to overall CFTR-dependent ion transport in the murine nasal epithelium.^7^ More recently, Zhang et al. demonstrated that PIV3-mediated *CFTR* delivery, which transduced ≥25% of airway epithelial cells and predominantly targeted ciliated cells, fully restored CFTR ion channel function in CF HAEC cultures.^4^

Two considerations are key to critically interpret these findings. First, the threshold of CFTR-expressing cells required to ameliorate CF lung disease was established when CFTR airway expression was attributed predominantly to ciliated cells—a notion disproven in 2018 and 2021 by the seminal single cell transcriptomics papers of Plasschaert,^8^ Montoro,^9^ and Okuda et al.^6^—revealing the historical lack of cell type resolution in analytical techniques employed at the time. Furthermore, the proposed thresholds stemmed from bulk functional assays, which intrinsically overlook cell-type-dependent differences in CFTR function in heterogeneous epithelia such as the airways. Therefore, to this date, it remains unclear whether all transduced cells contribute equally to functional rescue or a disproportionate impact is exerted by cell types that naturally express CFTR—e.g., secretory cells and ionocytes, the main contributors to total airway CFTR expression. These cells express specialized molecular machinery for CFTR biosynthesis, post-translational processing, and membrane trafficking, as well as the CFTR interactome contributing to ion transport coordination. Thus, CFTR restoration within such cell types may produce enhanced functional benefits and ultimately reduce the threshold of transduced cells and/or CFTR expression level per cell required to achieve therapeutic efficacy. Together, these considerations highlight the limitations of bulk functional readouts and underscore the need for functional single-cell approaches to identify target cells for future CF gene therapies and establish biologically meaningful correction thresholds.

Beyond efficient delivery, durability of transgene expression is crucial for *CFTR* gene therapy. McCray’s team reported a gradual decline in CFTR expression over 30 days, attributed to progressive dilution of the PIV5 AVLP genomes due to cell division rather than shedding of transduced cells due to cytotoxicity^2^—as had been instead the case for PIV3-based vectors.^4^ Wei et al., however, previously observed sustained GFP expression for over 6 weeks in the same conditions^5^—a surprising incongruency, which warrants investigation to exclude *CFTR*-transgene-specific silencing mechanisms.

Regular re-dosing would be required to offset the progressive decrease in CFTR expression and retain therapeutic benefit. In recent vaccine trials, pre-existing neutralizing antibodies did not hamper PIV5-mediated immunization, which suggests but cannot yet confirm that long-term repeated dosing might be well-tolerated and effective, in particular because anticipated vector titers for gene therapy are likely to be higher than those for immunization.^10^

Adequate prediction of immune responses to CF gene therapies remains challenging. Several gene therapy products that demonstrated safety and efficacy in animal models failed to translate into clinical success, underscoring the need for human-derived platforms. Current HAE models, however, are devoid of immune cells and therefore only recapitulate epithelial-cell-specific responses. The advent of novel, immunocompetent model systems, such as lung-on-a-chip and *ex vivo* lung perfusion models, pave the way to explore their validity in assessing efficacy and safety of novel gene therapy products in the pipeline. Adding further complexity, the CF airway is characterized by altered epithelial homeostasis, including a thickened mucus layer, aberrant immune responses, and chronic inflammation^2^—all potentially influencing efficacy and safety outcomes. Disease-relevant humanized models should therefore be prioritized to improve the predictive value of preclinical studies.

While the landscape of CF model systems requires reshaping, the PIV5 AVLP *CFTR* delivery platform proposed by Phillips, Lei et al. puts forward a very promising performance by restoring CFTR function to WT levels in state-of-the-art HAE models. If immune tolerability, potent and durable transgene expression, and the possibility of re-dosing can be confirmed in more complex CF models, a path toward clinical translation comes closer for this innovative CF gene therapy strategy.

## Acknowledgments

M.S.C. received project funding from the Cystic Fibrosis Foundation (CFF, CARLON24G0), Research Foundation Flanders (FWO, G0A9T25N), and the Italian Cystic Fibrosis Foundation (Fondazione per la Ricerca sulla Fibrosi Cistica, FFC#03/2025). K.G. is a beneficiary of the personal scholarship 1SA0O26N FWO-SB from Research Foundation Flanders. L.L.D. is a doctoral fellow, and M.S.C. a beneficiary of MSCA-DN GET-IN EC HORIZON–MSCA-2023-DN grant agreement no. 10111988.

## Declaration of interests

The authors declare no competing interests.
